# Therapeutic Re-Activation of Protein Phosphatase 2A in Acute Myeloid Leukemia

**DOI:** 10.3389/fonc.2015.00016

**Published:** 2015-02-02

**Authors:** Kavitha Ramaswamy, Barbara Spitzer, Alex Kentsis

**Affiliations:** ^1^Molecular Pharmacology and Chemistry Program, Department of Pediatrics, Sloan Kettering Institute, Memorial Sloan Kettering Cancer Center, Weill Medical College of Cornell University, New York, NY, USA

**Keywords:** protein phosphatase 2A, gene expression, enzyme activation, leukemia, kinase signaling

## Abstract

Protein phosphatase 2A (PP2A) is a serine/threonine phosphatase that is required for normal cell growth and development. PP2A is a potent tumor suppressor, which is inactivated in cancer cells as a result of genetic deletions and mutations. In myeloid leukemias, genes encoding PP2A subunits are generally intact. Instead, PP2A is functionally inhibited by post-translational modifications of its catalytic C subunit, and interactions with negative regulators by its regulatory B and scaffold A subunits. Here, we review the molecular mechanisms of genetic and functional inactivation of PP2A in human cancers, with a particular focus on human acute myeloid leukemias (AML). By analyzing expression of genes encoding PP2A subunits using transcriptome sequencing, we find that PP2A dysregulation in AML is characterized by silencing and overexpression of distinct A scaffold and B regulatory subunits, respectively. We review the mechanisms of functional PP2A activation by drugs such as fingolimod, forskolin, OP449, and perphenazine. This analysis yields two non-mutually exclusive mechanisms for therapeutic PP2A re-activation: (i) allosteric activation of the phosphatase activity, and (ii) stabilization of active holo-enzyme assembly and displacement of negative regulatory factors from A and B subunits. Future studies should allow the development of specific and potent pharmacologic activators of PP2A, and definition of susceptible disease subsets based on specific mechanisms of PP2A dysregulation.

## Introduction

Protein phosphatase 2A (PP2A) is a serine/threonine protein phosphatase that is required for normal cell growth and development and is frequently inactivated in human cancers (1). The functions of PP2A depend on its regulated phosphatase activities toward specific substrates, controlling kinase-dependent signal transduction, protein stability, cell proliferation, and survival (2). The functions of PP2A as a tumor suppressor in human cancers was originally defined as a result of its functional inactivation by the direct binding of the transforming antigens of the SV40 polyoma virus, as well as inactivating deletions and mutations of genes encoding its enzymatic subunits (3). Cells transformed by the polyoma T antigens or mutations of PP2A subunits exhibit reduced phosphorylation of PP2A substrates, including key mediators of mitogenic signaling, apoptosis, and mitosis; reviewed by Sabina et al. (4). Since normal cell growth and development are incompatible with complete inactivation of PP2A (5, 6), cell transformation induced by PP2A inactivation likely involves partial effects on specific substrates.

Specific recruitment of cellular substrates to the phosphatase catalytic subunit of PP2A is mediated by the assembly of its trimeric holo-enzyme (7). Active PP2A holo-enzyme is composed of the regulatory B, catalytic C, and scaffold A subunits, with the AC heterodimer forming the core enzyme (8). There is large diversity in the types of PP2A holo-enzymes formed, which is due to the array of genes and isoforms responsible for PP2A subunits with genetic redundancy of at least two genes encoding each of the different subunits. This diversity leads to heterogeneity in subunit isoforms in terms of genetic alterations, differential tissue expression, and varying types of malignancies. Pharmacological inhibition of PP2A phosphatase activity by toxins or small molecule drugs, including okadaic acid and microcystin, causes cellular transformation (9, 10), raising the possibility that pharmacological re-activation of residual PP2A activity may have anti-tumor efficacy. Here, we review the molecular mechanisms of PP2A in human cancer, with a specific focus on myeloid leukemias, and describe pharmacological strategies for its therapeutic re-activation.

## Genetic Mechanisms of PP2A Inactivation

Consistent with the requirement of PP2A activity for normal cell growth and development, genetic inactivation of PP2A appears to involve deletions and mutations of A or B subunits, but not the catalytic C subunit. The PP2A holo-enzyme is assembled by the A scaffold subunit, as structured by its HEAT repeat domain (11). In this way, the HEAT domain serves to juxtapose the regulatory B subunits that recruit specific substrates with the catalytic C subunit, as a result of protein–protein interactions of the N-terminal HEAT repeats 1–10 and C-terminal repeats 11–15 of the scaffold A subunit, respectively (12). The scaffold subunit A is expressed as constitutive and tissue-specific isoforms and encoded by different genes, PR65α (*PPP2R1A*) and PR65β (*PPP2R1B*), respectively with the PPP2R1B isoform responsible for 5–10% of the scaffold subunit (13). For example, breast and lung carcinomas inactivate PP2A by point mutations of the *PPP2R1A* gene and exon 9 deletion in the *PPP2R1B* genes, allowing for inactivation of PP2A phosphatase activity (14). Breast carcinomas also produce defective A subunits due to the deletions of amino acids 171–589. Similarly, deletion of E344–E388 that spans HEAT repeats 9 and 10 causes defective binding of specific regulatory B subunits in breast carcinomas. Mutations of the A scaffold PR65α also include mis-sense mutations E64G in breast carcinomas and R418W in melanomas, leading to the impaired recruitment of the B regulatory and C catalytic subunits, respectively (15). The PR65β A scaffold isoform appears to be more frequently affected by mis-sense mutations, including G8R, P65S, G90D, K343E, D504G, and V545A (16). Mutants of the PR65α isoform of subunit A have impaired binding to the B56γ B subunit, induce functional haploinsufficiency and contribute to cell transformation and near complete suppression of this Aα isoform leads to growth arrest (17).

In addition to the mutations of the A scaffold subunit, human cancers also frequently exhibit altered gene expression of the regulatory B subunits. For example, the *PPP2R2A* gene, which codes for the regulatory subunit B55α, is deleted in breast and prostate carcinomas and myelomas (18–20). In melanomas, there is reduced gene expression of the B56γ subunit encoded by *PPP2R5C* (21). In acute myeloid leukemias (AML), genes encoding PP2A subunits are generally intact, without apparent somatic mutations or deletions identified to date. One exception is the haploinsufficiency of the *PPP2CA* gene encoding the catalytic C subunit in AML cases with deletions of chromosome 5, including del(5q) specifically (contribution in this issue by Sallman et al.). The 5q commonly deleted region also includes several additional tumor suppressor genes (22), suggesting that haploinsufficiency of *PPP2CA* may cooperate with other gene deletions in AML.

**Figure 1:**
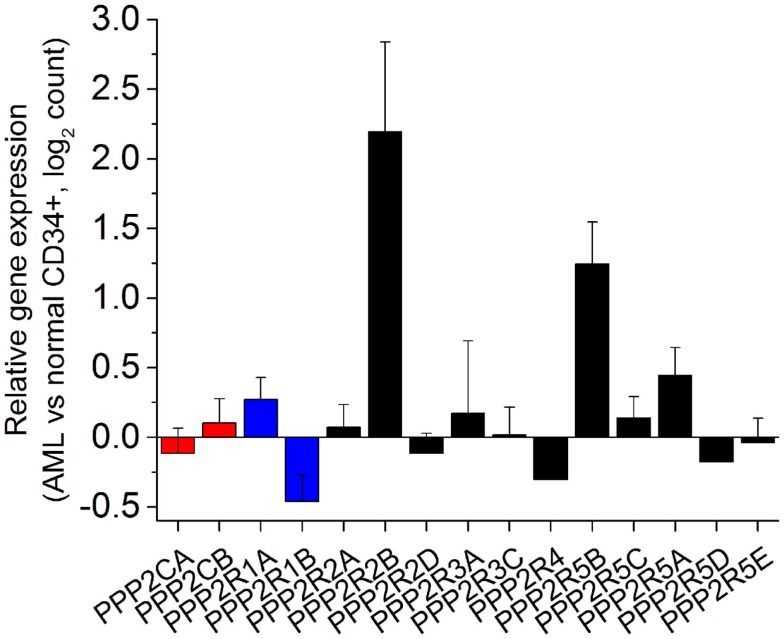
**Human acute myeloid leukemia cells exhibit distinct alterations in the expression of specific genes encoding PP2A subunits**. Relative expression of genes encoding PP2A subunits (catalytic C subunits in red, scaffold A subunits in blue, and regulatory B subunits in black) measured as a ratio of expression in AML cells relative to normal CD34+ bone marrow hematopoietic progenitor cells in normalized read counts. *PPP2R2C* and *PPP2R3B* are not significantly expressed and thus not shown, whereas *PPP2R1B, PPP2R2B*, and *PPP2R5B* are statistically significantly altered in their expression in AML cells relative to normal CD34+ cells (Benjamini Hochberg-adjusted *p* < 0.05).

Analysis of PP2A subunits and genetic mutations have until now focused at the level of genetic alteration and post-translational modifications. The correlation of these mutations with protein levels in AML cells of abnormal PP2A components has yet to be determined. In order to understand the expression of PP2A subunits in human AML, we analyzed transcriptome data collected as part of the Cancer Genome Atlas (TCGA) from 138 patients with AML and 5 healthy controls (23). Raw sequences were aligned to the hg19 human reference genome using STAR (24) and differential expression analysis was determined using DESeq2 Bioconductor package (25). As can be seen in Figure 1, most genes encoding PP2A subunits do not have statistically significantly altered expression in AML cells relative to normal CD34+ bone marrow precursor cells. We find that *PPP2R1B*, encoding the Aβ scaffold subunit, is significantly down-regulated in AML cells (Benjamini Hochberg-adjusted *p* = 0.036). This analysis also reveals the relative overexpression of genes encoding two regulatory B subunits in AML cells, *PPP2R2B* and *PPP2R5B* (Benjamini Hochberg-adjusted *p* = 0.0025 and 0.00021, respectively). Genes encoding regulatory B subunits *PPP2R2C* and *PPP2R3B* were found to be minimally expressed in AML cells in this analysis. In addition, *PPP2CA* encoding one of the catalytic C subunits is distinctly down-regulated in patients with *TP53* mutant AML, consistent with cytogenetically complex karyotypes in this disease subset, and with prior observations of its loss in del(5q) AML (26). We did not find any other apparent associations between PP2A subunit gene expression and molecular subtypes of AML in this analysis. Combined with the analysis of the expression of PP2A subunits in normal hematopoietic cells (contribution in this issue by Haesen et al.), these findings indicate that AML cells express a distinct repertoire of PP2A enzymes, characterized by down-regulation of scaffold A subunits and up-regulation of specific regulatory B subunits.

## Functional PP2A Inactivation

In addition to the apparent alterations in expression of PP2A enzymatic subunits, PP2A activity can be dysregulated through functional mechanisms, including post-translational modifications of the enzymatic C subunit and effects of regulatory proteins that interact with the A and B subunits. The PP2A catalytic C subunit is known to undergo phosphorylation and carboxymethylation, both of which have been found to regulate its enzymatic activity in the context of active holo-enzyme. Several oncogenic kinases, including BCR–ABL1 in myeloid leukemias, phosphorylate T304 and T307 of the C subunit, leading to reduced PP2A phosphatase activity (27, 28). Carboxymethylation of L309 of the C subunit by the methylesterases LCMT1 and PME-1 leads to altered recruitment of regulatory B55 subunits (29). Finally, oxidative nitration of Y289 of the B56δ subunit was recently described to interfere with the assembly of the PP2A holo-enzyme, leading to dysregulation of BCL-2 phosphorylation and aberrant cell survival in lymphoblastic leukemia and lymphoma cells (30). In summary, PP2A activity appears to be controlled by two non-mutually exclusive mechanisms: (i) allosteric regulation of the phosphatase activity, and (ii) regulation of holo-enzyme assembly and recruitment of specific substrates by the A and B subunits. These two mechanisms inform possible pharmacological strategies for therapeutic re-activation of PP2A, as discussed below.

In addition to regulatory post-translational modifications of PP2A, several endogenous cellular factors have been found to inhibit PP2A activity, including CIP2A and SET. Overexpression of these cellular inhibitors of PP2A leads to functional inactivation of the PP2A phosphatase activity, and contributes to cellular transformation. For example, CIP2A appears to transform cells by direct interaction with the c-Myc transcription factor and interfering with substrate recruitment by PP2A B subunits. In colon and head and neck squamous cell carcinomas, overexpression of CIP2A leads to the potentiation of MYC S62 phosphorylation, the PP2A target, thereby inhibiting proteolysis and resulting in increased oncogenic MYC protein stability (31, 32). Overexpression of CIP2A is also associated with increased phosphorylation of other PP2A substrates, including AKT1, MEK1, and MDM2, presumably due to interference with the assembly of additional PP2A holo-enzyme complexes targeting a variety of substrates (33).

Another well-characterized cellular inhibitor of PP2A is SET, and its regulator SETBP1. Although the exact mechanism is not well understood, SET is thought to inhibit PP2A by direct binding to the PP2A catalytic subunit (34). SET is overexpressed in breast carcinoma and acute leukemia cells, and can directly bind to the PP2A catalytic C subunit, causing reduced dephosphorylation of PP2A substrates (35). In AML, SET activity is regulated by proteolysis, mediated by its binding partner SETBP1, leading to the formation of an inhibitory SETBP1–SET–PP2A complex (36). When SETBP1 is overexpressed, SET is stabilized and protected from protease cleavage and facilitates PP2A inhibition, potentially explaining the relatively poor prognosis in patients with AML with altered expression of these regulatory proteins (37). In addition, some patients with AML exhibit SETBP1 overexpression due to the t(12;18)(p13;q12) chromosomal translocation (36), and patients with atypical CML can have recurrent G870S mutations of SETBP1 (38).

## Therapeutic Strategies to Reactivate PP2A

Absence of genetic mutations that cause complete loss-of-function of PP2A phosphatase activity, such as homozygous deletions of the C or A subunits and their genetic redundancy with two genes encoding these subunits, makes it possible to potentially reactivate PP2A for anti-cancer therapy. Such therapeutic PP2A re-activation strategies can be classified in two non-mutually exclusive mechanisms: (i) allosteric activation of the phosphatase activity, and (ii) stabilization of active holo-enzyme assembly and displacement of negative regulatory factors from A and B subunits. The C phosphatase subunit is known to be regulated by post-translational modifications of its C-terminal TPDYFL tail, including nitration of Y289, phosphorylation of T304 and Y307, and carboxymethylation of L309, which can potentially regulate the allosteric relay of the structurally juxtaposed 120–126 helix and 183–195 loop switches regulating the phosphatase catalytic site (39). Indeed, inhibition of PP2A by carcinogenic antibiotics such as okadaic acid and microcystin, involves changes in the allosteric regulation of the PP2A catalytic subunit (40). This raises the possibility that either natural products or small molecules can be used to pharmacologically modulate the PP2A C subunit to allosterically activate its phosphatase activity in the context of an active holo-enzyme (mechanism I). Indeed, forskolin, a diterpene antibiotic, can reduce the phosphorylation of the C-terminal tail PP2A C subunit Y307, leading to PP2A activation (41, 42). This effect appears to be independent of forskolin’s activation of adenylate cyclase, as evidenced by the apparently equal anti-leukemic efficacy of 1,9-dideoxy-forskolin, which does not alter cyclic AMP levels. Consistent with this, forskolin treatment was found to be synergistic with cytotoxic AML chemotherapeutic agents, idarubicin and cytarabine (41).

Fingolimod or FTY720, a derivative of the antibiotic myriocin, activates PP2A in part by inhibition of the negative PP2A regulator SET (mechanism II). Fingolimod can bind SET directly, causing its displacement from and assembly of active PP2A holo-enzymes (43). Treatment of AML cells with fingolimod leads to dephosphorylation of PP2A substrates, such as the KIT receptor, AKT1, and STAT5, causing cell death (44, 45). Because fingolimod also inhibits sphingosine kinase 1, current efforts are focused on using fingolimod derivatives that lack this activity, such as (R)-FTY720-OMe and OSU-2S to specifically activate PP2A (46). Similarly, the cell-penetrating peptide OP449 or COG449 is an apolipoprotein E-mimetic that directly binds SET, relieving SET-mediated PP2A phosphatase inhibition (47, 48).

Recently, perphenazine and related phenothiazines were found to activate PP2A, causing dephosphorylation of PP2A substrates and leukemia cell death (49). Perphenazine can bind directly to the PP2A Aα scaffold subunit (49). This can cause PP2A activation either due to the displacement of a negative A subunit regulator, such as CIP2A (31), and/or allosteric effects of the C subunit activity when bound to the A scaffold HEAT domains 11–15. This mechanism may also explain the pervasive inhibition of serine/threonine kinase signaling by the chemically related trifluoperazine drugs (50). Elucidation of the structural basis and effects on PP2A holo-enzyme assembly and phosphatase activity should lead to the development of more potent and specific phenothiazine-like PP2A activators.

## Future Therapeutic Strategies

Cellular PP2A comprises an ensemble of distinct holo-enzymes associated with specific regulatory subunits. Cancer cells with non-genetic mechanisms of PP2A inactivation, such as altered expression of genes encoding PP2A subunits or aberrant expression of PP2A negative regulators, may therefore be candidates for pharmacological functional re-activation of PP2A for anti-cancer therapy. In AML specifically, PP2A phosphatase activity appears to be dysregulated as a result of reduced expression of specific A scaffold subunits, and altered expression of regulatory B subunits. Consequently, leukemogenic effects of PP2A dysregulation are mediated by specific phospho-serine and phospho-threonine substrates. Likewise, pharmacological strategies for therapeutic PP2A re-activation will depend on the specific details of PP2A dysregulation. For example, pharmacologic inhibitors of PP2A negative regulator SET, such as fingolimod, OP449 and its analogs, will likely be ineffective in leukemias without SET overexpression or activation. In contrast, pharmacological PP2A activators that modulate PP2A phosphatase activity through allosteric regulation of phosphatase activity, such as forskolin and possibly perphenazine and related drugs, may have broader efficacy. Finally, detailed understanding of the molecular mediators of carcinogenic PP2A dysregulation, including specific substrates and signaling pathways, will be necessary to define not only biomarkers that can be used to prospectively identify susceptible tumors but also to determine effective combination therapies.

## Conflict of Interest Statement

The authors declare that the research was conducted in the absence of any commercial or financial relationships that could be construed as a potential conflict of interest.
